# Cost-effective lignocellulolytic enzyme production by *Trichoderma reesei* on a cane molasses medium

**DOI:** 10.1186/1754-6834-7-43

**Published:** 2014-03-24

**Authors:** Jun He, Ai-min Wu, Daiwen Chen, Bing Yu, Xiangbing Mao, Ping Zheng, Jie Yu, Gang Tian

**Affiliations:** 1 Institute of Animal Nutrition, Sichuan Agricultural University, Ya’an, Sichuan 625014, People’s Republic of China; 2 College of Forestry, South China Agricultural University, Guangzhou 510642, People’s Republic of China

**Keywords:** Enzyme, *Trichoderma reesei*, Cane molasses, Carbon source, Proteomics

## Abstract

**Background:**

Cane molasses, an important residue of the sugar industry, have the potential as a cost-effective carbon source that could serve as nutrients for industrial enzyme-producing microorganisms, especially filamentous fungi. However, the enzyme mixtures produced in such a complex medium are poorly characterized. In this study, the secretome of *Trichoderma reesei* grown on a cane molasses medium (CMM) as well as on a lactose-based conventional medium (LCM) were compared and analyzed by using proteomics.

**Results:**

In this study we show that both the CMM and LCM can serve as excellent growth media for *T. reesei*. The enzyme expression patterns in the two media were similar and a considerable number of the identified proteins on two-dimensional gel electrophoresis (2-DE) gels were those involved in biomass degradation. The most abundant cellulolytic enzymes identified in both media were cellobiohydrolases (Cel7A/Cel6A) and endoglucanases (Cel7A/Cel5A) and were found to be more abundant in CMM. We also found that both media can serve as an inducer of xylanolytic enzymes. The main xylanases (XYNI/XYNIV) and xyloglucanase (Cel74A) were found at higher concentrations in the CMM than LCM.

**Conclusions:**

We analyzed the prevalent proteins secreted by *T. reesei* in the CMM and LCM. Here, we show that hydrolytic enzymes are cost-effective and can be produced on cane molasses as a carbon source which can be used to digest lignocellulolytic biomass.

## Introduction

Lignocellulose is the most abundant renewable carbon resource on earth. It is synthesized mainly by plants and is the main constituent of plant cell walls. In recent years, more emphasis has been made to produce bioethanol from lignocelluloses derived from non-food sources and its use as an alternative to fossil fuels [1]. However, the high cost of its production remains one of the major obstacles for its cost-effective production [2,3]. Hydrolyzing enzymes (cellulase and hemicellulase) that are used to convert lignocellulosic polysaccharides to fermentable sugars contribute substantially to the cost of bioethanol production.

The filamentous fungus *Trichoderma reesei* is a well-known producer of lignocellulolytic enzymes that are used for depolymerization of plant lignocellulosic biomass [4]. Systematic strain improvement by mutagenesis and screening has resulted in several industrial strains producing over 100 g/L extracellular proteins, with approximately half of the secreted proteins consisting of the main cellulase, cellobiohydrolase I (Cel7a) [5].

Previous studies indicated that the production levels and enzyme systems (the relative proportions of different hydrolases) of *T. reesei* are influenced to some extent by varying the compositions of media and growth conditions [6-8]. For instance, lactose, which is a conventional carbon substrate used in industrial production media, not only promotes good growth but also strongly induces the expression of cellulase genes [9,10]. However, the utilization of xylose or maltose as the carbon source significantly alters the composition of the secreted enzymes by *T. reesei*[10,11]. Cane molasses are an important residue of the sugar industry, consisting of approximately 50% (w/w) total sugars (mainly sucrose, glucose and fructose), water, crude protein and fat, heavy metals, vitamins and other nutrients [12]. Although cane molasses with appropriate treatment were found to be a suitable nutrient source for a number of industrial microorganisms, including *T. reesei*[12-15], the enzyme mixtures produced in such media are not well characterized with respect to protein identification and quantification.

In the present study, the secretome of *T. reesei* Rut C-30 (a strain already used at industrial-scale) grown on a cane molasses medium (CMM) and on a lactose-based conventional medium (LCM) was analyzed by using two-dimensional gel electrophoresis (2-DE). Proteins of interest were identified by using matrix-assisted laser desorption/ionization mass spectrometry (MALDI-MS) and electrospray ionization liquid chromatography tandem mass spectrometry (ESI-LC MS/MS). To the best of our knowledge, this is the first report on the secretome of *T. reesei* in CMM. Moreover, this study offers a basis for further investigation of cost-effective enzyme production using industrial residues.

## Results and discussion

### Growth of *T. reesei* in CMM and LCM

**Figure 1:**
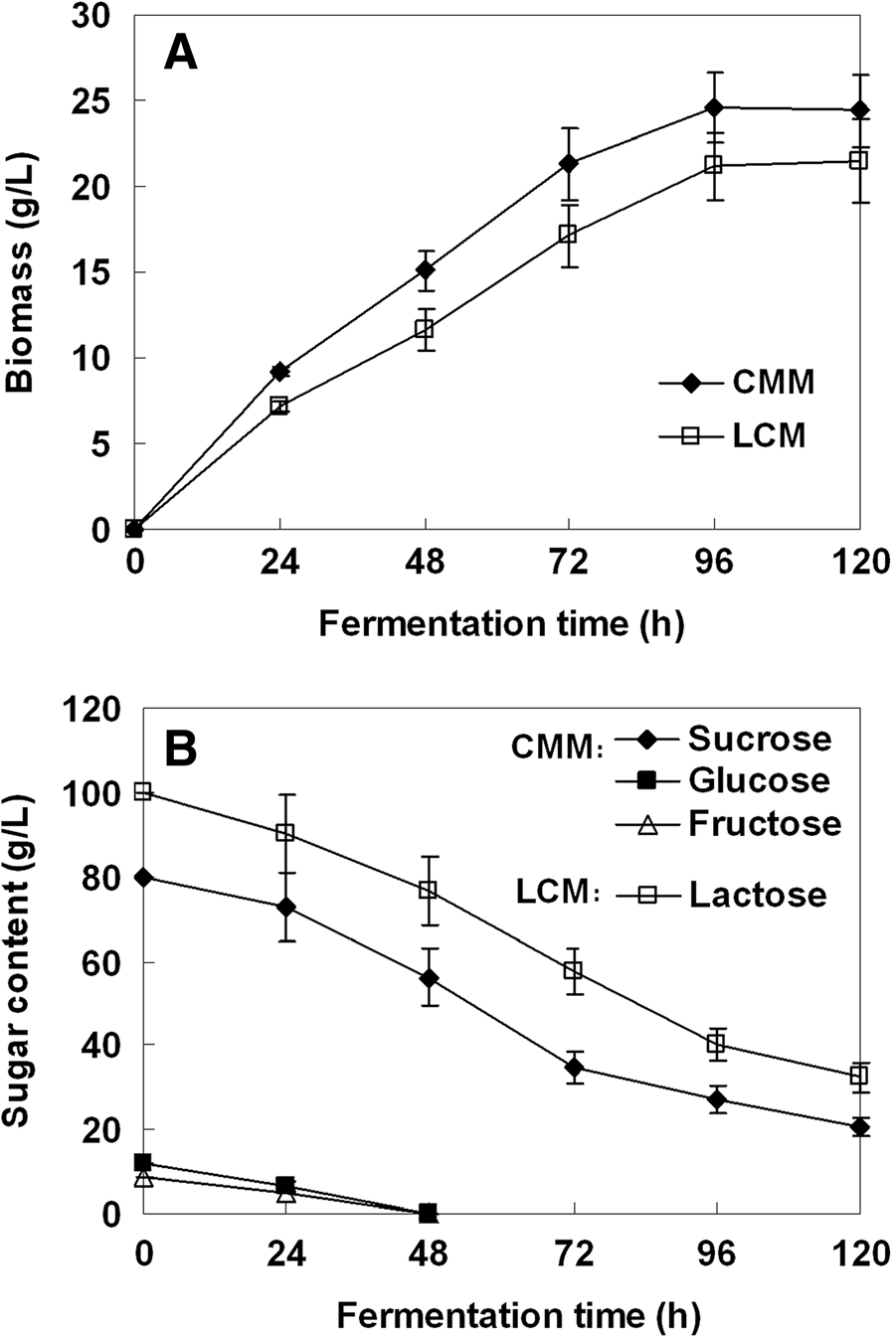
**Biomass production and sugar consumption by *T. reesei *Rut C-30 grown on LCM and CMM. (A)** Biomass production; and **(B)** sugar consumption. CMM, cane molasses medium; LCM, lactose-based conventional medium.

Before fermentation, crude molasses were diluted with distilled water to obtain 10% (w/v) total sugar concentration and centrifuged to remove ash and other undissolved impurities. Therefore, the CMM consists of 79.8 g/L sucrose, 11.7 g/L glucose and 8.5 g/L fructose. Our results show that both the CMM and LCM (containing 10% lactose) can serve as an excellent growth medium for *T. reesei* (Figure 1A). However, the CMM yielded the highest biomass formation with 25.4 g/L, despite the fact that lactose is one of the most favored carbons by *T. reesei*[9,10]. The sugar concentration was recorded throughout the fermentation process and the results show that some sugars still remained unfermented in the two media when samples for secretome analysis were taken (Figure 1B). We found that both the glucose and fructose in CMM were depleted after 48 hours of fermentation. This result is in good agreement with the fact that both of the two sugars are a readily usable carbon source for microbial growth, but would act as a repressor of cellulase synthesis in *T. reesei*[16,17].

### Enzyme production by *T. reesei* in CMM and LCM

The production of the main lignocellulolytic enzymes by *T. reesei* is transcriptionally regulated and carbon source-dependent [18-20]. In the present study, cultivation of *T. reesei* in CMM resulted in a higher total filter paper activity (FPase) and activity on carboxymethyl cellulose (CMCase). The β-glucosidase (EC 3.2.1.21), catalyzing the hydrolysis of cellobiose to glucose, was also present at an elevated level in the CMM (Table 1). This finding is interesting considering the widespread hypothesis that the expression of the main cellulase genes in *T. reesei* is antagonized by the presence of the preferred carbon sources such as the glucose and fructose in the CMM [16,17]. Our results are, however, consistent with previous reports that the *cre1* deficient strain Rut C-30 used in this study does not down-regulate gene expression of cellulases and hemicellulases on easily metabolized sugars [16,17]. Moreover, we found that xylanolytic enzymes were produced in both media, and the total xylanase activity produced in CMM was 135% higher than in the LCM (172.1 versus 73.1 U/mL). The higher xylanase activity observed in CMM was probably due to the presence of sucrose, since it was found to be a stronger inducer of xylanase than lactose in *Thermomyces lanuginosus*[21].

**Table 1:** **Specific enzyme activities produced by *T. reesei *** **in CMM and LCM**

**Enzyme activity**	**CMM**	**LCM**
Biomass^a^ (DW g/L)	25.4 ± 2.09	22.5 ± 2.45
Protein content^b^ (mg/mL)	4.69 ± 0.05	4.25 ± 0.04
FPase (U/mg protein)	0.94 ± 0.11	0.51 ± 0.09
CMCase (U/mg protein)	1.15 ± 0.21	0.64 ± 0.11
β-glucosidase (U/mg protein)	3,948 ± 79	1,270 ± 35
Xylanase (U/mg protein)	36.7 ± 4.7	17.2 ± 1.9

^a^Biomass production by *T. reesei* at the end of fermentation; ^b^protein content in the culture supernatant at the end of fermentation. CMCase, activity on carboxymethyl cellulose; CMM, cane molasses medium; FPase, filter paper activity; LCM, lactose-based conventional medium.

### Two-dimensional gel electrophoresis (2-DE) map of the *T. reesei* secretome

**Figure 2:**
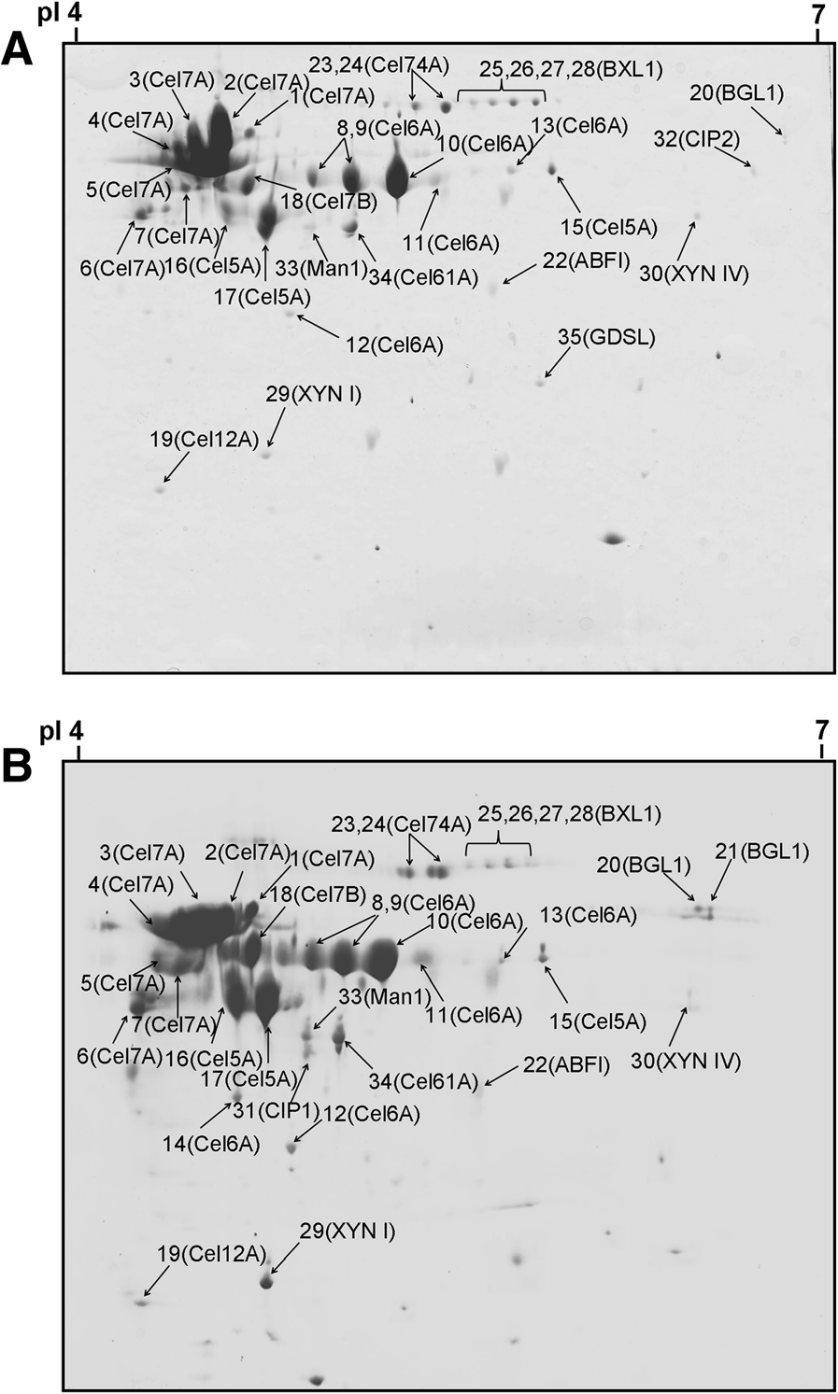
**2-DE analysis of secreted proteins by *T. reesei *Rut C-30 grown on LCM or CMM. (A)** LCM; and **(B)** CMM. The identified protein spots are labeled by the protein abbreviations given in the Table 2. 2-DE, two-dimensional gel electrophoresis; CMM, cane molasses medium; LCM, lactose-based conventional medium.

Previous studies have indicated that enzymes produced by *T. reesei* are media-dependent [18-20]. The enzymes produced from different types of complex media may give completely different patterns of enzyme production. 2-DE is a powerful tool to visualize hundreds of proteins at a time, which in combination with mass spectrometry offers a way to identify them. In order to obtain the fullest complement of the hemicellulolytic enzymatic system, a lactose-based reference medium (LCM) was used, since lactose is known to induce the production of both cellulases and hemicellulases in *T. reesei*[22,23]. The protein maps are shown in Figure 2. We found a total of 46 distinct protein spots on the 2-D gel after staining. The distribution of the protein spots shows that most of the extracellular proteins have an isoelectric point (pI) below 6 and a molecular mass above 20 kDa. Through MALDI-MS and ESI-LC MS/MS analysis, 35 protein spots were identified as enzymes relating to the degradation of lignocellulosic biomass (Table 2). The types of identified lignocellulolytic enzymes were more than we obtained from a batch fermentation in flask [10]. The results clearly show the advantage of using *T. reesei* Rut C-30 as a most suitable microbial strain for lignocellulolytic enzyme production [4,5].

**Table 2:** **Identification by MALDI-MS and ESI-LC MS/MS of proteins secreted by *T. reesei *** **Rut C-30 grown in CMM and LCM**

**Number**	**ProtID**^**a**^	**Protein name**	**Precursor mass (kDa)**	**pI**^**b**^	**Mascot score**^**c**^	**Peptides matched**	**Sequence coverage (%)**
1^d^	123989	Cellobiohydrolase I (Cel7A)	55.4	4.6	73	5	13
2^d^	123989	Cellobiohydrolase I (Cel7A)	55.4	4.5	73	5	13
3^d^	123989	Cellobiohydrolase I (Cel7A)	55.4	4.5	73	5	13
4^d^	123989	Cellobiohydrolase I (Cel7A)	55.4	4.5	73	5	13
5^d^	123989	Cellobiohydrolase I (Cel7A)	55.4	4.5	73	5	13
6^e^	123989	Cellobiohydrolase I (Cel7A)	54.1	4.4	598	21	24.9
7^e^	123989	Cellobiohydrolase I (Cel7A)	54.1	4.5	201	8	16.7
8^d^	72567	Cellobiohydrolase II (Cel6A)	50.3	4.8	107	10	27
9^d^	72567	Cellobiohydrolase II (Cel6A)	50.3	4.9	100	9	27
10^d^	72567	Cellobiohydrolase II (Cel6A)	50.3	5.0	83	8	26
11^d^	72567	Cellobiohydrolase II (Cel6A)	49.6	5.1	133	5	13.4
12^e^	72567	Cellobiohydrolase II (Cel6A)	35.1	4.7	471	22	21.4
13^e^	72567	Cellobiohydrolase II (Cel6A)	49.6	5.5	94	5	6.6
14^e^	72567	Cellobiohydrolase II (Cel6A)	39.2	4.6	135	8	9.2
15^d^	120312	Endoglucanase II (Cel5A)	48.7	5.8	56	4	24
16^e^	120312	Endoglucanase II (Cel5A)	44.1	4.5	340	23	30.4
17^e^	120312	Endoglucanase II (Cel5A)	44.1	4.7	219	5	18.4
18^e^	122081	Endoglucanase I (Cel7B)	48.2	4.6	106	4	9.2
19^d^	123232	Endoglucanase III (Cel12A)	25.1	4.5	83	8	32
20^d^	76672	β-Glucosidase I (BGLI)	78.7	6.4	196	20	31.2
21^d^	76672	β-Glucosidase I (BGLI)	78.7	6.5	106	17	23.6
22^d^	123283	Arabinofuranosidase (ABFI)	40.2	5.5	113	11	25.1
23^d^	49081	Xyloglucanase (Cel74A)	87.3	5.2	184	15	29
24^d^	49081	Xyloglucanase (Cel74A)	87.3	5.4	123	11	24.7
25^d^	121127	β-Xylosidase (BXLI)	87.5	5.5	188	21	41
26^d^	121127	β-Xylosidase (BXLI)	87.5	5.6	132	15	24.1
27^d^	121127	β-Xylosidase (BXLI)	87.5	5.7	101	14	26.3
28^d^	121127	β-Xylosidase (BXLI)	87.5	5.8	155	17	28.6
29^e^	74223	Xylanase I (XYNI)	24.6	4.7	142	3	9.4
30^d^	111849	Xylanase IV (XYNIV)	53.4	5.9	139	10	24
31^e^	73638	Cellulose-binding protein (CIPI)	32.9	4.8	317	13	20.3
32^d^	123940	Cellulose-binding protein (CIPII)	49.0	6.3	107	13	29
33^d^	56996	Mannanase I (MANI)	40.1	4.8	97	8	19.2
34^d^	73643	Endoglucanase IV (Cel61A)	40.2	4.9	86	2	6.1
35^d^	121418	Lipolytic enzyme (GDSL)	39.3	5.7	113	11	34

^a^From The Genome Portal of the Department of Energy Joint Genome Institute (JGI) for *T. reesei*; ^b^pI determined by 2-DE; ^c^analyzed by ^d^MALDI-MS or ^e^ESI-LC MS/MS. 2-DE, two-dimensional gel electrophoresis; CMM, cane molasses medium; ESI-LC MS/MS, electrospray ionization liquid chromatography tandem mass spectrometry; LCM, lactose-based conventional medium; MALDI-MS, matrix-assisted laser desorption/ionization mass spectrometry.

We noted that the enzyme production patterns in the two growth media were qualitatively similar (Figure 2). In both media, the cellobiohydrolases Cel7A and Cel6A were the most abundantly secreted proteins. The higher intensity of the corresponding protein spots observed on the 2-D gels was consistent with previous findings that the two proteins account for 70 to 80% of the total *T. reesei* cellulase [24,25]. We also identified four out of the five known endoglucanases (Cel7B, Cel5A, Cel12A and Cel61A) on the 2-D gels (Table 2). One endoglucanase (Cel45A) was not identified, which may be due in part to being produced only in a low amount and having a highly acidic pI [25]. Similar to previous reports, we only identified a β-glucosidase on 2-D gels as BGL1 [10,26]. This is probably due to the fact that other β-glucosidases are either intra-cellular, membrane-anchored, or play only a minor role in cellulose hydrolysis [27].

Further, some major components of the hemicellulolytic system of *T. reesei*, such as β-xylosidase (BXL1), xylanase (XYN), xyloglucanase (Cel74A) and arabinofuranosidase (ABFI), were also identified on the 2-D gels (Table 2). Surprisingly, only two xylanases, XYNI and XYNIV, were detected on the 2-D gels. The XYNII which has been considered as one of the major xylanases secreted by *T. reesei* was not detected [28]. Moreover, the galactosidases which catalyze the hydrolysis of galactosides into monosaccharides were not identified in both media. This is also surprising, since these enzyme proteins were found to be produced in a lactose-based medium [29]. However, it may be possible that these proteins corresponded to one of the minor unidentified spots. In addition to cellulases and hemicellulases, some non-hydrolytic proteins such as CIPI and CIPII were also identified. Both these two proteins were previously characterized without a glycosyl hydrolase functional domain except for a cellulose-binding domain [19]. However, they share close relationships with cellulases and may have an important role in biomass degradation [30].

### Quantification and comparison of the extracellular protein produced by *T. reesei*

**Figure 3:**
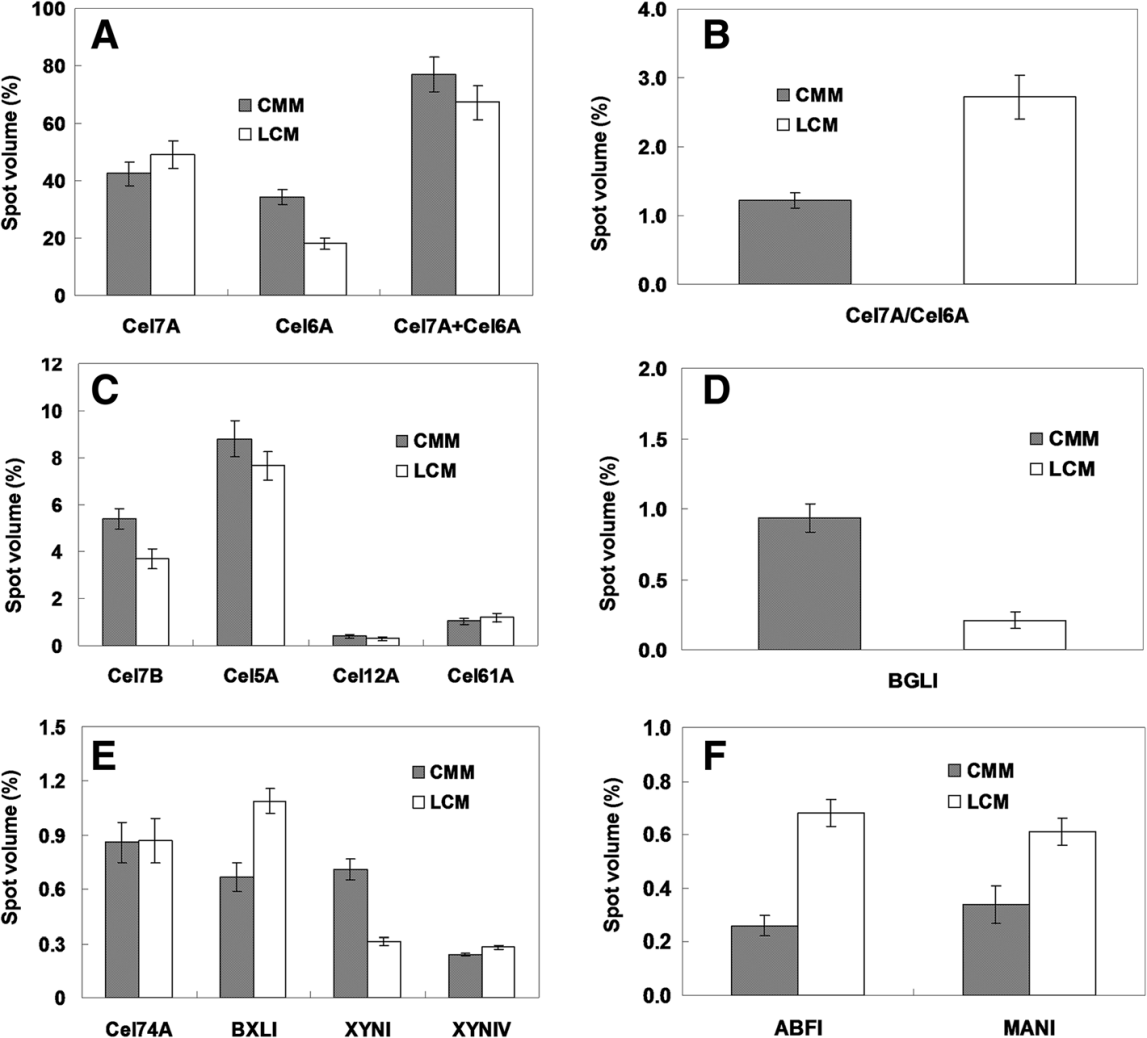
**Comparative analysis of the secretome of *T. reesei *Rut C-30 grown on LCM or on CMM. (A)** Relative abundance of Cel7A and Cel6A; **(B)** Cel7A to Cel6A ratio; **(C)** relative abundance of Cel7B, Cel5A, Cel12A and Cel61A; **(D)** relative abundance of β-glucosidase; and **(E,F)** relative abundance of other identified hemicellulolytic enzymes. CMM, cane molasses medium; LCM, lactose-based conventional medium.

The relative amount for each protein spot (percentage of total spots volume) was quantified using ImageMaster II software (GE Healthcare, Uppsala, Sweden). The spot numbers detected on the two protein maps were similar. However, *T. reesei* secreted more enzyme proteins into the CMM than LCM. It is a well-known fact that *T. reesei* is an efficient producer of cellulolytic enzymes, and expresses two cellobiohydrolases (Cel7A and Cel6A), enzymes that catalyze the release of cellobiose from the reducing or non-reducing end of cellulose, and five endoglucanases (Cel7B, Cel5A, Cel12A, Cel61A and Cel45A), enzymes that attack cellulose in an endo-acting manner [31]. Our protein spot quantification indicated that *T. reesei* produced 9.7% more total cellobiohydrolase (Cel7A + Cel6A) in the CMM than LCM (Figure 3A). This is due to a significantly higher Cel6A level produced in the CMM (34.5% in CMM versus 18.1% in LCM). As a result, the ratio of Cel7A to Cel6A was much higher in the LCM than CMM (Figure 3B). This finding is surprising since the expression of Cel7A and Cel6A was considered to be co-regulated [32]. Similarly, the relative amount of total identified endoglucanases (Cel7B + Cel5A + Cel12A + Cel61A) produced was also higher in CMM (Figure 3C). These results are, however, not only consistent with the carbon-dependent nature of the cellulolytic system in *T. reesei*, but also suggest that the sugars present in the CMM (sucrose) may be more efficient than lactose as an inducer for the main cellulolytic enzymes.

Complete degradation of cellulosic materials requires an exocellulase with specificity for a variety of β-D-glycoside substrates [33]. β-Glucosidase (EC 3.2.1.21), catalyzing the hydrolysis of terminal non-reducing residues in β-D-glucosides with release of glucose, are usually induced by specific substrates such as lactose [10]. Other carbon substrates, such as xylose, arabinose and sophorose (one of the most potent inducers of *T. reesei* cellulases), do not induce β-glucosidase (BGL1) and, in high concentrations, even repress its formation [10,34]. In the present study, the enzyme was induced in both media, and the total amount of BGL1 produced in the CMM was 4.5-fold higher than that in the LCM (Figure 3D). Previous studies have indicated that BGL1 may be involved in the formation of cellulase inducers [27,35]. Therefore, the elevated total cellulase activity in the CMM may result from a higher BGL1 level produced in this medium.

*T. reesei* is also found to be an efficient producer of hemicellulases. The relative amount of β-xylosidase (BXL1) required for complete degradation of xylan to xylose was higher in the LCM than in CMM (Figure 3E). The result is consistent with a previous report that lactose is a stronger BXL1 inducer than sucrose and fructose in *Cellulomonas flavigena*[36]. Moreover, the glucose in the CMM was, however, considered as a non-inducing sugar [36,37]. No significant difference was observed with respect to the level of Cel74A and XYNIV in the two growth media. However, both the level of XYNI and the total xylanase (XYNI + XYNIV) were found at elevated levels in the CMM (Figure 3E). The other two *T. reesei* xylanases (XYNII and XYNIII) were not detected, which could be because they were produced in lower amounts and have a basic pI (the predicted pI of XYNII is 7.9) that is out of the pH range (pH 4 to 7) used in the 2-DE analysis. We also cannot rule out the possibility that they have a higher level of degradation in the two media [10,26]. The relative amount of other identified hemicellulases, such as the arabinofuranosidase (ABFI) and mannanase (MANI), also showed marked differences. However, these two enzymes were produced only in small amounts (Figure 3F).

While the results reported were reproducibly obtained on all 2-D gels analyzed, it should be noted that some spots might have contained co-migrating proteins that were not detected in the MALDI-MS analysis. These proteins would have affected the relative quantification of the identified proteins. As MALDI-MS identifies the prevalent proteins that are present in a gel sample, these errors are, however, considered to be negligible. Moreover, it is noteworthy that the relative amount (spot volume) for identified enzyme proteins fit well with data obtained from specific enzyme activity (Table 1). The higher FPase and CMCase in the CMM can be related to the higher amounts of cellobiohydrolases (Cel7A + Cel6A) and endoglucanases (Cel7B + Cel5A + Cel12A + Cel61A) in this medium. Similarly, the higher β-glucosidase and xylanase-specific activities are consistent with the higher amounts of BGL1 and total xylanases (XYNI + XYNIV) produced in the CMM.

## Conclusion

Both the CMM and LCM can serve as an excellent growth medium for *T. reesei*. As compared to the LCM, *T. reesei* produces a considerable number of cellulases in the CMM and also higher levels of xylanases. Importantly, this study not only shows the prevalent proteins secreted by *T. reesei* in the CMM and LCM, but the results also suggest that production of hydrolytic enzymes using cost-effective carbon sources, such as components in cane molasses, deserves more attention in the future.

## Methods

### Pretreatment of cane molasses

Cane molasses were obtained from Meishan sugar refinery (Sichuan, People’s Republic of China). It contained 27% (w/w) water, 37.5% (w/w) sucrose, 9.5% (w/w) reduced sugars (glucose and fructose), 2.5% (w/w) other carbohydrates, 4.5% (w/w) crude protein, 0.1% (w/w) crude fat, 8.3% (w/w) ash, 4.1% (w/w) salt and 6.5% (w/w) metal ions, such as calcium, potassium, sodium, iron, magnesium, copper, and so on. The crude molasses were diluted with distilled water to obtain 10% (w/v) total sugar concentration and centrifuged to remove ash and other undissolved impurities. This molasses solution was used for the pretreatment of cation exchange resin (to remove metal ions) as described by Roukas [38].

### Fermentation in multi-bioreactor

Batch cultivations were carried out in a bioreactor (Runze Biotech Co. Ltd., Nanjing, People’s Republic of China) using *T. reesei* Rut C-30. Six 500 mL bioreactor vessels (three vessels per group) equipped with condensers, FermProbe pH electrodes and dissolved oxygen sensors (pO_2_ electrodes) were autoclave-sterilized and filled with 250 mL pretreated cane molasses (CMM) or a reference medium (with 10% lactose as the carbon substrate) [18]. Foam was suppressed by the addition of 0.1% ergosterol/Tween 80 mixture (consisting of 10 g/L ergosterol and 420 g/L Tween 80) and eight drops of antifoam. Each bioreactor vessel was inoculated with *T. reesei* spores to yield an initial cell density of 1 × 10^6^ spores/mL. Throughout the fermentation, the temperature was kept at 30°C, the stirring was kept at 300 rpm and the pH was kept at 5.5. The fermentation was aborted after 5 days. The protein samples (culture supernatant) were collected by centrifugation for 15 minutes at 12,000 *g* and 4°C.

### Protein extraction and 2-DE

The proteins in the supernatant were precipitated and purified by using a 2-D Clean-Up Kit (GE Healthcare). The purified protein sample was dissolved in rehydration solution (4% 3-((3-cholamidopropyl) dimethylammonio)-1-propanesulfonate (CHAPS), 8 M urea and 0.002% bromophenol blue) supplemented with 2% (v/v) 4-7 IPG Buffer (GE Healthcare) and 2.8 mg/mL dithiothreitol. Regardless of the initial protein concentration in the culture supernatant, the same amount of protein was used for each 2-D gel. Immobiline DryStrip gels (18 cm, pH 4 to 7; GE Healthcare) were rehydrated overnight at room temperature with 300 μg of protein dissolved in rehydration solution (about 400 μL). Isoelectric focusing (IEF) was performed with a Multiphor II system (GE Healthcare) at 20°C with a three-phase gradient program: 500 V for 0.25 kVh, 3,500 V for 5.25 kVh and 3,500 V for 28 kVh. Following IEF, each strip was equilibrated for 12 minutes in 10 mL of SDS equilibration buffer (50 mM Tris-HCl, 6 M urea, 30% (v/v) glycerol, 2% (w/v) SDS and 0.002% bromophenol blue) containing 1% (w/v) dithiothreitol. A second equilibration step was then performed with 2.5% (w/v) iodoacetamide added to the SDS equilibration buffer. 2-DE was performed on a PROTEAN™ II electrophoresis system (Bio-Rad, Hercules, CA, USA). The immobilized pH gradient (IPG) strips were placed on top of 12.5% polyacrylamide gels and sealed with a solution of 1% (w/v) agarose containing a trace of bromophenol blue. The vertical gels were run at 10 mA per gel for 30 minutes followed by 25 mA per gel until the bromophenol blue had migrated to the bottom of the gel. The temperature was maintained at 15°C using a MultiTemp III system (GE Healthcare). After electrophoresis, the gels were stained with SYPRO Ruby Protein Gel Stain (Invitrogen, Carlsbad, CA, USA).

### Image analysis

For comparative analysis, each culture sample was independently prepared and used in 2-DE analyses performed in triplicates. The SYPRO ruby-stained gels were scanned at 532 nm using a Typhoon 9400 scanner (GE Healthcare). Images were analyzed using ImageMaster II software. After automatic spot detection, artifacts, such as dust on gels, were manually removed and the weaker spots (<0.1% of the whole gel volume) were eliminated. The remaining spots were then automatically linked to reference spots on a synthetic reference gel (containing a 1:1 mixture of the protein extract from the two culture supernatants) to allow normalization and comparison of samples. The normalized volume of spots on three replicate 2-D gels was averaged and the standard deviation was calculated.

### In-gel digestion and mass spectrometry

For mass spectrometry analysis, protein spots were picked from the 2-D gels using an Ettan Spot Picker station (GE Healthcare) and distained three times using a fresh solution of 20 mM ammonium bicarbonate containing 35% (v/v) acetonitrile. Subsequently, the gel pieces were dried by two washes using 100% neat acetonitrile and rehydrated on ice using a solution of sequencing grade modified trypsin (Promega, Madison, WI, USA) in 20 mM ammonium bicarbonate. The trypsin concentration depended on the intensity of the spots and was 2 to 3 ng/μL. The rehydrated gel samples were incubated in 37°C for overnight digestion. MALDI-MS spectra for peptides were acquired using a Voyager-DE STR mass spectrometer (AB SIEX, Stockholm, Sweden) as described by Yao *et al*. [39]. LC-MS/MS combined with ESI-ion-trap MS was performed using an HCT-Ultra ETD II mass spectrometer from Bruker (Bremen, Germany) linked to an Easy-nLC system from Proxeon (Odense, Denmark). Spectra were acquired using the enhanced scanning mode covering a mass range from m/z 300 to m/z 1,300. The LC separation of peptides was performed using a 5 μm C18 column (375 μm OD/75 μm ID × 10 cm) from NanoSeparations (Nieuwkoop, The Netherlands) and a 30-minute gradient ranging from 0 to 60% of acetonitrile. The flow rate was 300 nL/min^−1^. Database searches using the peak list files of the processed mass spectra were performed using an in-house license of Mascot (Matrix Science Inc., Boston, MA, USA; http://www.matrixscience.com) and the Genome Portal of the Department of Energy Joint Genome Institute (JGI, Walnut Creek, CA, USA; http://www.jgi.doe.gov) for *T. reesei*.

### Enzymatic assays

Enzymatic activities were measured in culture supernatants obtained after centrifugation. Overall cellulase activity of the samples was determined as FPase using Whatman No. 1 filter paper strips as the substrate [40]. Endoglucanase activity was measured as CMCase with carboxymethyl cellulose (CMC) dissolved in 50 mM citrate buffer (pH 5.0) as the substrate. The assay was performed for 30 minutes at 50°C. Xylanase activity was assayed by the method described by Bailey *et al*. [41]. Oat spelt xylan (Sigma-Aldrich, St Louis, MO, USA) was used as the substrate. The amount of released sugar was assayed via the dinitrosalicylic acid (DNS) method using glucose or xylose as the standard [42]. β-Glucosidase activities were determined using 4-nitrophenyl-β-D-glucopyranoside with para-nitrophenol as the standard [43]. One unit (U) of enzyme activity was defined as the quantity of enzyme that liberated substrate at the rate of 1 μmol per minute.

### Biomass and chemical analysis

To determine the microbial biomass production (cell dry weight), the *T. reesei* cells were harvested continuously through filtration. Harvested cells were washed and dried at 80°C until constant weight was achieved. The monosaccharide content in the CMM and LCM after fermentation was analyzed by anion exchange chromatography using a DX-500 system (Dionex, Sunnyvale, CA, USA) equipped with a CarboPac PA1 column (Dionex) [44].

## Abbreviations

2-DE: Two-dimensional gel electrophoresis; ABFI: Arabinofuranosidase; BGLI: β-Glucosidase I; Cel5a: Endoglucanase II; Cel6a: Cellobiohydrolase II; Cel7a: Cellobiohydrolase I; Cel7b: Endoglucanase I; Cel12a: Endoglucanase III; Cel61a: Endoglucanase IV; Cel74a: Xyloglucanase; CHAPS: 3-((3-cholamidopropyl) dimethylammonio)-1-propanesulfonate; CIPI: Cellulose-binding protein; CMC: Carboxymethyl cellulose; CMCase: Activity on carboxymethyl cellulose; CMM: Cane molasses medium; DNS: Dinitrosalicylic acid; ESI-LC MS/MS: Electrospray ionization liquid chromatography tandem mass spectrometry; FPase: Filter paper activity; IEF: Isoelectric focusing; IPG: Immobilized pH gradient; JGI: Joint Genome Institute; LCM: Lactose-based conventional medium; MALDI-MS: Matrix-assisted laser desorption/ionization mass spectrometry; MANI: Mannanase I; pI: Isoelectric point; XYN: Xylanase; XYNI: Xylanase I; XYNIV: Xylanase IV.

## Competing interests

The authors declare that they have no competing interests.

## Authors’ contributions

HJ and MX carried out the sample preparation and the 2-DE analyses. WA, YJ and TG carried out in-gel digestions and chemical analyses. CD, YB and ZP performed the mass spectrometric analyses and database searches. All authors substantially contributed to the analysis and the drafting of the manuscript. All authors read and approved the final manuscript.
